# Targeting TGF-β/VEGF/NF-κB inflammatory pathway using the Polyphenols of *Echinacea purpurea* (L.) Moench to enhance wound healing in a rat model

**DOI:** 10.1007/s10787-025-01681-6

**Published:** 2025-03-07

**Authors:** Marwa I. Ezzat, Mai M. Abdelhafez, Asmaa K. Al-Mokaddem, Shahira M. Ezzat

**Affiliations:** 1 Pharmacognosy Department, Faculty of Pharmacy, Cairo University, Kasr El-Ainy Street, Cairo, 11562 Egypt; 2 Department of Pharmacology and Toxicology, Faculty of Pharmacy, October University for Modern Sciences and Arts (MSA), 6Th October, 12451 Egypt; 3 Department of Pathology Faculty of Veterinary Medicine, Cairo University, Giza, 12211 Egypt; 4 Department of Pharmacognosy, Faculty of Pharmacy, October University for Modern Sciences and Arts (MSA), 6Th October, 12451 Egypt

**Keywords:** Polyphenols of *Echinacea* *purpurea*, UPLC/ESI, MS, Molecular networking, Wound, Healing, Inflammation, TGF, β/VEGF/NF, κB

## Abstract

The present study explores the metabolic profiling and molecular wound-healing mechanisms of *Echinacea* *purpurea* (L.) Moench (EP) flowers aqueous (AE) and ethanol (EE) extracts in an excision wound-healing model. Metabolic profiling of the extracts was investigated using UHPLC-ESI-TOF–MS and molecular networking. Antioxidant activity was carried out using the DPPH (1, 1-diphenyl-2-picrylhydrazyl) radical scavenging method and FRAP (ferric reducing antioxidant power). Carboxy methylcellulose gels of 5 and 10% of both aqueous (AE) and ethanol (EE) extracts were prepared. The wounds were explored macroscopically, histologically, and immunohistochemically. The UHPLC-ESI-TOF–MS method enabled the identification of 3 organic acids, 14 phenolic acids, 3 phenylethanoid glycosides, and 11 flavonoids from EP extracts. EE had significant antioxidant activity compared to AE. The EP treated wounds healed faster. The EE succeeded in improving healing properties and controlling the inflammatory response by reducing IL-6 and increasing IL-10 expression and enhancing angiogenesis and remodeling via increased NF-κB, TGF-β, VEGF, CD31 expression and α-SMA and collagen deposition. It is worth mentioning that the EE groups also showed improvement in the histopathological examination in a dose-dependent manner. The effectiveness of EE in wound-healing may be attributed to its higher content of polyphenols which also made the antioxidant potential of the EE and its capacity to donate electrons higher than that of AE. This study scientifically enables the understanding of the molecular mechanisms *Echinacea* *purpurea* extract in wound healing via modulating skin inflammatory response and indicates the potential usefulness of *EP* ethanol extract for wound healing.

## Introduction

Skin is the most important organ that acts as a protective barrier against harmful agents and external stimuli. A wound is a break in the integrity of the epithelium and may cause harm to the underlying normal tissue's function and structure (Masson-Meyers et al. 2020; Mulkalwar et al. 2015).

The process of restoring the normal structure and functions of injured tissues through wound healing is a complicated biological and molecular one that requires the synthesis of matrix components, biomolecular interactions, migration, proliferation, interaction, and differentiation of various cell types (such as epidermal, dermal, and infiltrating inflammatory cells), as well as a complex signaling network (Stephens et al. 2013).

Healthy endothelial cells are essential to restore normal tissues after injury (Johnson and Wilgus TAJAiwc. 2014). They play an important role in two essential steps in the process of wound healing: inflammation and angiogenesis (Wilkinson and Hardman MJJOb. 2020). Delayed wound healing can result in several complications that interfere with the patient’s quality of life and increase healthcare costs until the affected patients are fully recovered (Sa and DiPietro LAJJodr. 2010).

Inflammation is the second stage of the healing process which requires the local viable vasculature to allow inflammatory cells to be recruited to the site of injury. The damaged endothelial cells stimulate the innate response to clear the wounded area from debris and foreign bodies (Chen and DiPietro LAJAiwc. 2017). The infiltration by pro-inflammatory cells triggers the release of many cytokines such as IL-1beta and tumor necrosis factor-α (TNF-α) via activation of the NF-κB (nuclear factor-κB) signaling pathway (Kolaczkowska and Kubes PJNri. 2013). The healthy surrounding vasculature is crucial to help the recruitment of several immune cells including neutrophils, macrophages, fibroblasts, as well as endothelial cells (Li et al. 2007). At a later stage during wound healing, these cells are secreting additional cytokines to suppress inflammation and start the angiogenesis phase via production of tissue growth factor-β (TGF-β) and vascular endothelial growth factor (VEGF). The formation of new vasculature is thereby triggered and later maintained by suppressing endothelial cell apoptosis via upregulating anti-apoptotic proteins such as BCL-2 (Cai et al. 2003).

Debridement, irrigation, tissue grafts, antibiotics, and proteolytic enzyme treatments are used for healing wounds; however, these methods have harmful side effects (Niederstätter et al. 2021). Topical treatments with antibiotics are selected based on their ability to inhibit the growth of harmful organisms since chronic wounds may harbor bacteria. Furthermore, anti-inflammatory medications such as corticosteroids and nonsteroidal anti-inflammatory drugs (NSAIDs) are used to stop the inflammatory effects of wounds (Xia et al. 2022). Early application of these medications, as a topical preparation, decreases the risk of systemic side effects, increases the concentration of the drug in the affected area, and offers the benefit of quantifiable drug usage (Teshome et al. 2022).

The previously mentioned conventional medications are effective, but they also have drawbacks such as bacterial resistance, side effects, costs, and inappropriate usage (Dai et al. 2019).

Finding an alternative, safe, and effective treatment has become critical. From the few available sources, herbs have been the main source used for developing new medications (Oguntibeju OOJVw. 2019). Plant-derived extracts and/or isolates work through a variety of interconnected processes to promote tissue regeneration and healing. Many of these are cost-effective and have minimal adverse side effects (Maver et al. 2018). Plant-derived polyphenols have been reported to be among the most popular compounds that have the potential to function as antibacterial, antioxidant, and anti-inflammatory agents during the wound healing process (Korkina et al. 2012).

*Echinacea purpurea (L.)* Moench (EP) is a perennial herbaceous flowering plant. It belongs to the Asteraceae family and commonly known as purple conical flower. It is native to North America and widely distributed in the United States (Burlou-Nagy et al. 1244).

EP extracts were traditionally used in North America for the treatment of wounds and different types of infections (Banica et al. 2020).

One of the most popular herbal preparations in both Europe and the United States is made using EP. *E. purpurea* had been introduced to China as a medicinal herb at the end of the previous century (Manayi et al. 2015). EP is a well-known natural medication that is relatively safe with immunomodulatory, anti-inflammatory, and antioxidant effects (Gu et al. 2023). Previous phytochemical studies indicated the presence of three groups of phytochemicals such as phenolic compounds (phenylethanoid glycoside, hydroxycinnamic acid derivatives, and flavonoids) polysaccharides and lipophilic alkylamides, which are responsible for the medicinal properties (Burlou-Nagy et al. 1244). Several in vitro and in vivo studies revealed that alkylamides are implicated in the immunomodulatory properties of *E. purpurea* extracts. Moreover, the anti-inflammatory effect of *E. purpurea* preparations is due to the presence of polysaccharides. Hydroxycinnamic derivatives and phenolic compounds play an important role in the antioxidant and anti-inflammatory effects of the plant (Matthias et al. 2007; Oláh et al. 2017; Chiou et al. 2017). The present study characterized the metabolic profiles of the extracts using UHPLC-ESI-TOF–MS and investigated the healing molecular mechanisms of skin after topical application of the ethanol and aqueous extracts of *E. purpurea* in a rat model of excision wound healing. The wounds were explored macroscopically, histologically, and immunohistochemically.

## Materials and methods

### Chemicals, reagents, and materials

Ammonium formate and acetonitrile were provided by (Sigma-Aldrich, Germany). Deionized water of LC–MS-grade was obtained from (Millipore, USA). Methanol, sodium hydroxide, and formic acid, ACS grade, were acquired from Fisher Scientific, USA. The chemicals and all other reagents were purchased from Sigma Chemical Co. (USA).

### Plant material and preparation of the plant extracts

The flowers of *E. purpurea* were collected in April 2022, from the Horticulture Research Center, Ministry of Agriculture, Giza. The identity was verified by Dr. Rim Hamdy, Assistant Professor of Plant Taxonomy & Flora, Faculty of Science, Cairo University. A voucher specimen (6–04-2022) was kept in the herbarium of Pharmacognosy Department, Faculty of Pharmacy, Cairo University, Cairo, Egypt.

The flowers of *E. purpurea* were subjected to size reduction after being air-dried in a normal atmosphere to produce coarse powder. The dried powder (500 g) was extracted sequentially with 70% ethanol and water by cold maceration until complete exhaustion. The ethanol extract (EE) was evaporated under reduced pressure at a temperature not exceeding 40 °C to yield 82.3 g. The aqueous extract (AE) was lyophilized to yield 58.5 g. The dry extracts were kept at − 20 °C for further study.

### UHPLC-ESI-TOF–MS analysis and metabolite identification

Sample preparation, acquisition method, and data processing are described in detail in our previous publication (Abbas et al. 2023).

### Molecular networking

To create the online workflow, the ProteoWizard MSConvert Version 3 software’s program was used to convert the RAW files into the open source “mzXML” file format. The mzXML files were uploaded using WinSCP, the recommended FTP client, to the GNPS online platform (https://gnps.ucsd.edu/ProteoSAFe/static/gnps-splash.jsp). The MN parameters were minimum pairs cosine, 0.65; precursor ion mass tolerance, 2 Da; fragment ion mass tolerance, 0.5 Da; min matched fragment ions, 6; and a minimum cluster size of 1. For processing, analysis, and visualization, network files were loaded into Cytoscape 3.10.0 (https://cytoscape.org/download.htm l), an open-source platform (Wang et al. 2016).

### Evaluation of antioxidant activity

#### DPPH radical scavenging method

DPPH free radical scavenging activity of the aqueous and ethanolic extracts were investigated using the method carried out by Boly et al. (2016). Briefly, 100 µL of a 0.1% solution of DPPH in methanol was added to 100 µL of each extract solution at different concentrations, followed by vigorous shaking of the mixture, which was then allowed to stand at room temperature for 30 min. Then the absorbance was measured at 540 nm in FluoStar Omega microplate reader. Microsoft Excel® was used to analyze the data and Graph Pad Prism 5® was utilized to determine the IC_50_ value by logarithmizing the concentrations and selecting the non-linear inhibitor regression equation (log (inhibitor) vs. normalized response—variable slope equation) (Chen et al. 2013). A higher free radical scavenging activity was shown by a lower absorbance of the reaction mixture.

DPPH scavenging effect (%) = 100 − [((A_0_-A_1_)/A_0_) × 100].

### Ferric reducing antioxidant power assay (FRAP)

The ferric reduction capacity of the EE and AE was estimated using a slightly modified method of Benzei and Strain (Benzie and Strain 1996). Ten μL of samples (EE, AE and Trolox) were mixed with 190 μL of freshly prepared TPTZ reagent (300 mM acetate buffer (PH = 3.6), 10 mM TPTZ in 40 mM HCl, and 20 mMFeCl_3_, in a ratio of 10:1:1 v/v, respectively). The absorbance was measured for 60 min in kinetic mode at 37 °C, at 593 nm. The FluoStar Omega microplate reader was used to record the results. The antioxidant activity was tested three times in order to assess the assays' repeatability. A linear regression equation for standard Trolox was used to get the FRAP values (µM TE/mg).

### Gel formulation of the extracts

Carboxymethylcellulose (CMC) was acquired from Sigma-Aldrich and according to the manufacturer’s recommendation CMC hydrogel was prepared. CMC can be used as a carrier for drugs and is reported to be compatible as a wound dressing (Kanikireddy et al. 2020). The method of *E. purpurea*—CMC gels preparation is as follows. A final CMC concentration of 5% was prepared by dissolving CMC in distilled water by constant stirring. Finally, the 5% and 10% (w/w) CMC gels of both the ethanol and aqueous extracts were prepared by integrating 5 g and 10 g of the crude extract into a 100 g of CMC hydrogel, respectively.

### In vivo experimental design

All animals were treated according to the MSA-IACUC guideline for animal extermination, the Egyptian national guideline and ARRIVE guideline. The rats were housed and the experiment was conducted in the MSA-animal facility. The animal experimentation was revised and approved by the Faculty of Pharmacy, Cairo University, approval number MP (3033).

Twenty adult male albino rats, weighing 150 ± 10 gm, were purchased from the MSA-University supplier. The animals were divided into five equal groups, named as the following: Control wounded group (treated with saline), ***E****chinacea purpurea* **E**thanol extract **L**ow dose (5% w/w, **EEL**), ***E****chinacea purpurea* **E**thanol extract **H**igh dose (10% w/w, **EEH**), ***E****chinacea purpurea* **A**queous extract **L**ow dose (5% w/w, **EAL**) and ***E****chinacea purpurea* **A**queous extract **H**igh dose (10% w/w, **EAH**).

### Excision wound healing model

As previously described by Bakr., et al. 2021, the animals were anesthetized using ketamine/xylazine mixture (80 mg/kg–8 mg/kg, respectively). Two excision wounds were induced on the dorsal back of rats after hair removal using a 1-cm^2^ biopsy punch. All the wounds were pictured using a digital camera and then treated according to the assigned group. The pictures and the treatment were repeated as follows (days 0, 2, 4, 6, and 9).

On the 9th day, the animals were euthanized and the healed wounded tissue was dissected. The samples from each wound were divided into half. One half was kept in formalin buffer (10%) for histological evaluation and the second half was homogenized using lysis buffer, centrifuged for 20 min at 20,000 rpm. The supernatant was isolated and stored at -80 °C for further biochemical analysis. Alternatively, the tissue was treated with TRIzol reagent Mini kit (Invitrogen, cat: 15,596–026), where RNA extracted from tissue homogenate was eluted in 45 µl in nuclease free distilled water and stored at -20 °C for PCR quantification.

### Wound healing rate analysis

The wound area was calculated from the pictures using Image J software (version 1.51) (Schneider et al. 2012). The healing percentage was calculated using the following equation: (100 – ((wound area at day 2, 4, 6 or 9/wound area at induction day) *100)).

### Histopathology, evaluation of wound healing criteria and collagen content

After dissection of skin samples at the wound area, the tissues were kept in formalin buffer (10%) for fixation. A routine tissue processing protocol was followed to prepare hematoxylin and eosin (H&E) stained sections for light microscopy. Tissue slides were examined and histological wound healing criteria were evaluated on a scale from 0 to 4 including re-epithelization, granulation tissue formation, inflammation and angiogenesis as previously described in Refai et al. (2023) with a modification in inflammation score that increased with increasing intensity. For evaluation of collagen content at the wound area, Masson’s trichrome stain (MTC) was used and the stained collagen was quantified as area percent.

### Immunohistochemistry

Positively charged glass slides were used to mount tissue sections for immune staining. Tissue sections from each group were incubated with primary anti-α-SMA, TGF-β, CD-31, VEGF, and TNF-α at a dilution of 1:100 for 1 h at room temperature followed by washing and detection steps using a HRP-labelled detection kit (Bio SB, USA) following manufacturer’s instructions. Positive expression was quantified as area percent using LAS-X software (Leica, Germany).

### Biochemical analysis (ELISA and qRT-PCR)

C3, C5b-9, IL-6 and IL-10 were measured in the supernatant of the homogenized healed skin tissue, using the commercially available kits and according to the manufacturer instructions. Rat complement 3 (C3, AFG Bioscience, cat: EK720727, USA), terminal complement complex (C5b-9, AFG Bioscience, cat: EK720861, USA), IL-6 (BioVision, cat: K4145-100, USA) and IL-10 (CUSBIO, cat: CSB-E04595r, USA) sandwich ELISA kits were applied.

Quantification of DNA expression of NF-κB was carried out using a NF-κB PCR fluorescence quantitative diagnostic kit SYBR green PCR master mix (SNP Biotechnology R&D, cat: BSA09S7, Turkey).

### Statistical analysis

Sample size calculation was done using G*Power software version 3.1.9 (Faul et al. 2009). Unless mentioned in the figure ligand, the statistical analysis was done using one-way ANOVA followed by Bonferroni multiple comparison test utilizing the GraphPad software version 9. The statistical analysis is expressed as mean ± standard deviation and significance considered when p-value is less than 0.05.

## Results

### UHPLC-ESI-TOF–MS analysis and metabolite identification

**Fig. 1:**
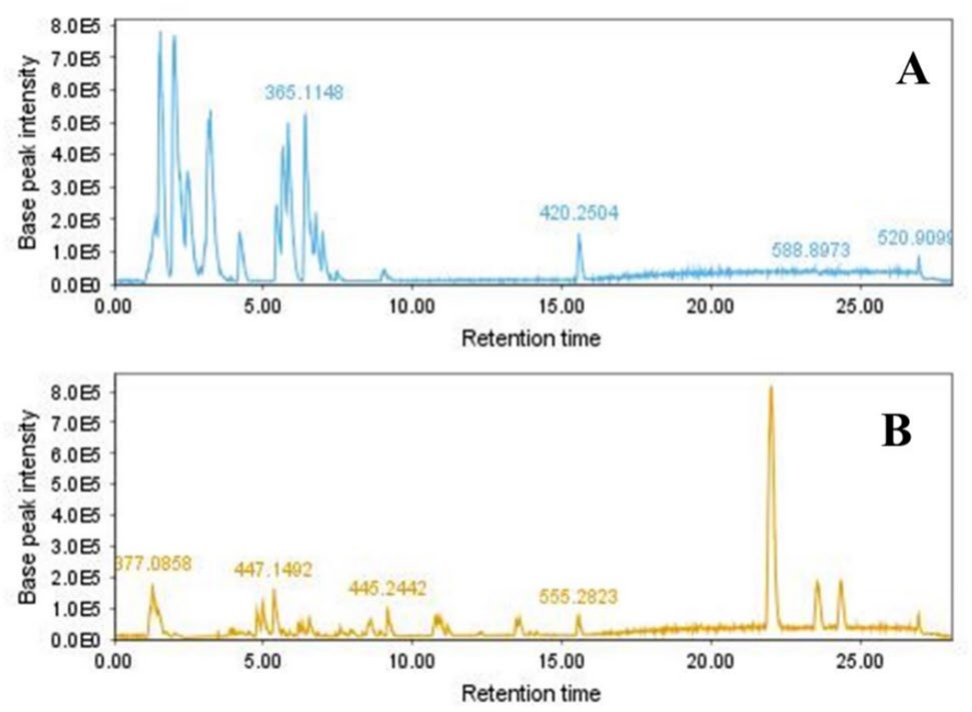
Base peak chromatograms from UHPLC-ESI–MS of *E. purpurea* extracts, aqueous (A), and ethanol (B) samples

The analysis of phytochemical profiles of ethanol and aqueous extracts was performed to identify potential variations in metabolites between samples. The representative base peak chromatograms of the two extracts in negative mode are shown in Fig. 1**.** The process of identifying and characterizing compounds involved comparing their molecular weights, MS spectra, and retention times using reliable databases like Dictionary of Natural Products and PubChem, as well as relevant literature. Table 1 reports the tentatively identified compounds in ethanol and aqueous extracts in negative ionization mode. Thirty-one compounds were identified from ethanol and aqueous extracts including 3 organic acids, 14 phenolic acids, 3 phenylethanoid glycosides, and 11 flavonoids.

**Table 1:** Bioactive secondary metabolites tentatively identified in ethanol and aqueous extracts of *E. purpurea* using UHPLC-ESI–MS analysis

Compound No	R_t_	Precursor ion [M-H]^−^	Name	Molecular formula	Fragments MS product ion	EE	AE	References
Organic acids								
	1.179	133.013	D- ( +)-Malic acid	C_4_H_6_O_5_	133	✓	✓	Refai et al. 2023)
	1.22	191.031	Quinic acid	C_7_H_12_O_6_	191, 173	✓	✓	Refai et al. 2023)
	1.27	149.045	Tartaric acid	C_4_H_6_O_6_	149	✓	✓	Faul et al. 2009)
Phenolic acids								
	1.23	311.077	Caftaric acid	C_13_H_12_O_9_	179, 149, 135	✓	✓	Refai et al. 2023; Coelho et al. 2020)
	1.35	473.01	Chicoric acid	C_22_H_18_O_12_	311, 179, 161, 149, 135	✓	✓	Refai et al. 2023; Faul et al. 2009; Coelho et al. 2020)
	1.42	179.15	Caffeic acid	C_9_H_8_O_4_	135	✓	✓	Refai et al. 2023)
	1.55	353.088	Chlorogenic acid	C_16_H_18_O_9_	191,179, 135	✓	✓	Coelho et al. 2020)
	2.57	337.092	3-*p*-Coumaroylquinic acid	C_16_H_18_O_8_	191, 163, 173		✓	Eldin et al. 2021)
	3.21	457.072	Coumaroylcaffeoyltartaric acid	C_22_H_18_O_11_	295, 163, 149		✓	Coelho et al. 2020)
	3.23	367.102	Methyl chlorogenic acid (3-O-Caffeoylquinic acid methyl ester)	C_17_H_20_O_9_	191, 173	✓	✓	Faul et al. 2009)
	4.677	359.185	Rosmarinic acid	C_18_H_16_O_8_	179, 161	✓	✓	Faul et al. 2009)
	5.36	515.487	Cynarin (1, 3-dicaffeoylquinic acid)	C_25_H_24_O_12_	353, 235, 179	✓	✓	Faul et al. 2009; Sharif et al. 2021)
	5.377	515.134	4, 5-Dicaffeoylquinic acid	C_25_H_24_O_12_	353, 335, 172		✓	Faul et al. 2009; Coelho et al.^2020^; Sharif et al. 2021)
	6.5	499.123	3-O-caffeoyl-5-O-*p*-coumaroylquinic acid	C_25_H_24_O_11_	353, 335, 172		✓	Faul et al. 2009)
	13.57	295.213	Coutaric acid (trans-*p*-Coumaroyl tartaric acid)	C_13_H_12_O_8_	163, 149, 119	✓	✓	Faul et al. 2009)
	22.27	399.275	Cinnamoylepoxyechinadiol	C_24_H_32_O_5_	131, 119	✓		Faul et al. 2009)
	23.58	255.232	1-O-Dihydrocaffeoylglycerol	C_12_H_16_O_6_	181, 180, 163, 161, 137, 136	✓	✓	Faul et al. 2009)
Phenylethanoid glycosides								
	4.6	445.172	Echipuroside A	C_20_H_30_O_11_	164, 146, 137	✓	✓	Faul et al. 2009; Karamać et al. xxxx)
	10.75	785.354	Echinacoside	C_35_H_46_O_20_	623, 461, 315, 179	✓	✓	Faul et al. 2009)
	15.54	623.27	Verbascoside	C_29_H_36_O_15_	461, 315, 179	✓		Faul et al. 2009)
Flavonoids								
	3.447	269.138	Apigenin	C_15_H_10_O_5_	225,149,107	✓	✓	Faul et al. 2009)
	4.55	491.162	Tricin 7-glucoside	C_23_H_24_O_12_	329, 313	✓	✓	Park et al.^2012^,
	5.014	301.035	Quercetin	C_15_H_10_O_7_	151,179	✓		Refai et al. 2023)
	5.3	315.108	Isorhamnetin (3-methylquercetin)	C_16_H_12_O_7_	301, 272, 256, 164, 151	✓		Faul et al. 2009)
	5.36	447.15	Astragalin (Kaempferol-3-O-glucoside)	C_21_H_20_O_11_	285,284, 255	✓	✓	Faul et al. 2009)
	6.55	329.124	Tricin (5,7,4’-trihydroxy-3’,5’-dimethoxyflavone)	C_17_H_14_O_7_	329, 314, 299, 271	✓	✓	Faul et al. 2009)
	6.895	593.16	Nicotiflorin (Kaempferol 3-O-rutinoside)	C_27_H_30_O_16_	285, 255	✓	✓	Refai et al. 2023; Faul et al. 2009; Clifford et al. 2005)
	7.568	609.149	Rutin (Quercetin-3-O-rutinoside)	C_27_H_30_O_16_	301, 179, 151	✓	✓	Coelho et al. 2020; Clifford et al. 2005)
	7.728	623.153	Isorhamnetin-3-O-rutinoside	C_28_H_32_O_16_	315, 314, 300, 257, 151	✓	✓	Refai et al. 2023)
	14.14	577.267	Apigenin Xylopyranosyl-glucuronopyranoside	C_30_H_26_O_12_	457, 413, 293	✓		Faul et al. 2009)
	14.53	595.287	Quercetin 3-O-β-D-glucopyranoside, 7-O-D-xyloside	C_26_H_28_O_16_	577, 565, 445, 415	✓	✓	Faul et al. 2009)

### Organic acids

Tartaric acid, malic acids, and quinic acid were identified in EE and AE. Fragmentation patterns of the identified compounds were compared to reference literature as shown in Table 1.

### Phenolic acids and their derivatives

Several phenolic acids, and their derivatives were tentatively identified (10 phenolic acids in EE and 13 phenolic acids in AA), caffeic acid and its derivatives were the most abundant phenolic acids.

Chicoric acid was detected with [M-H]^−^ at *m/z* 473.01 producing the fragmentation pattern *m/z* 179 (caffeic acid fragment) and *m/z* 135 (caffeic acid—CO_2_) (Coelho et al. 2020; Eldin et al. 2021).

The caftaric and coutaric acids were identified by their [M—H] ions at *m/z* 311.077 and 295.213, respectively. Neutral loss of the tartaric acid moiety yielded the corresponding [M—H] ions of caffeic, and coumaric acids (Coelho et al. 2020; Eldin et al. 2021; Sharif et al. 2021). Chlorogenic acid and its derivative methyl chlorogenic acid showed the [M–H] at *m/z* 353.088 and *m/z* 367.102, respectively. They were identified by the presence of fragments at m/z 191 (deprotonated quinic) and 135 (decarboxylated caffeic acid) corresponding to which was consistent with previous reports (Eldin et al. 2021; Sharif et al. 2021). Two dicaffeoylquinic acid isomers (**12** and **13**) were detected (*m/z* 515). Both compounds showed a product ion at *m/z* 353 after loss of one caffeic acid moiety. Compounds **12** on further fragmentation showed a predominant fragment at *m/z* 179 representing the caffeic acid fragments, whereas fragmentation of compound **13** led to the formation of a fragment at *m/z* 172 (quinic acid–H_2_O). These results allowed the identification of compounds **12** and **13** as cynarin (1, 3-dicaffeoylquinic acid) and 4,5-di-*O*-caffeoylquinic acid, respectively (Eldin et al. 2021; Sharif et al. 2021).

### Phenylethanoid glycosides

Three compounds were identified in EE, two of them only were detected in AE. Compounds **19** and **20** with [M-H]^−^ ion at *m/z* 785.354 and 623.27 were identified as echinacoside and verbascoside, respectively. They exhibited a product ion at *m/z* 461 after loss of 162 amu that was attributed to the neutral loss of caffeoyl moiety. The further loss of 146 amu gave the fragment ion at *m/z* 315 which was attributed to the loss of rhamnose moiety from the ion at *m/z* 461. The cleavage of the caffeoyl moiety produced the diagnostic ions at *m/z* 179 (Eldin et al. 2021).

### Flavonoids

Flavonoids were the major metabolites identified (11 in EE and 8 in AE), there were three flavonoid aglycone nuclei and 8 *O*-glycosides. Flavonoid-*O*-glycoside often show the product ion of their aglycones after initial loss of a sugar moiety. Compounds **27, 28** and **29** showed a loss of 308 Da (rutinoside moiety) to give the aglycone peak at *m/z* 285, 301, 315, respectively. Compounds **27**, **28** and **29** were identified as nicotiflorin (Kaempferol 3-O-rutinoside), rutin (Quercetin-3-O- rutinoside) and isorhamnetin-3-O-rutinoside, respectively. Compound **22** showed a [M − H] ion at m/z 491.162 and a high intensity of fragment ions at *m/* 329 after loss of a glucose molecule, representing the tricin fragment, and 313 [M–H–162–18] by the loss of the hydroxyl group. This compound was identified as tricin 7-glucoside.

### Molecular networking-based classification of metabolites

**Fig. 2:**
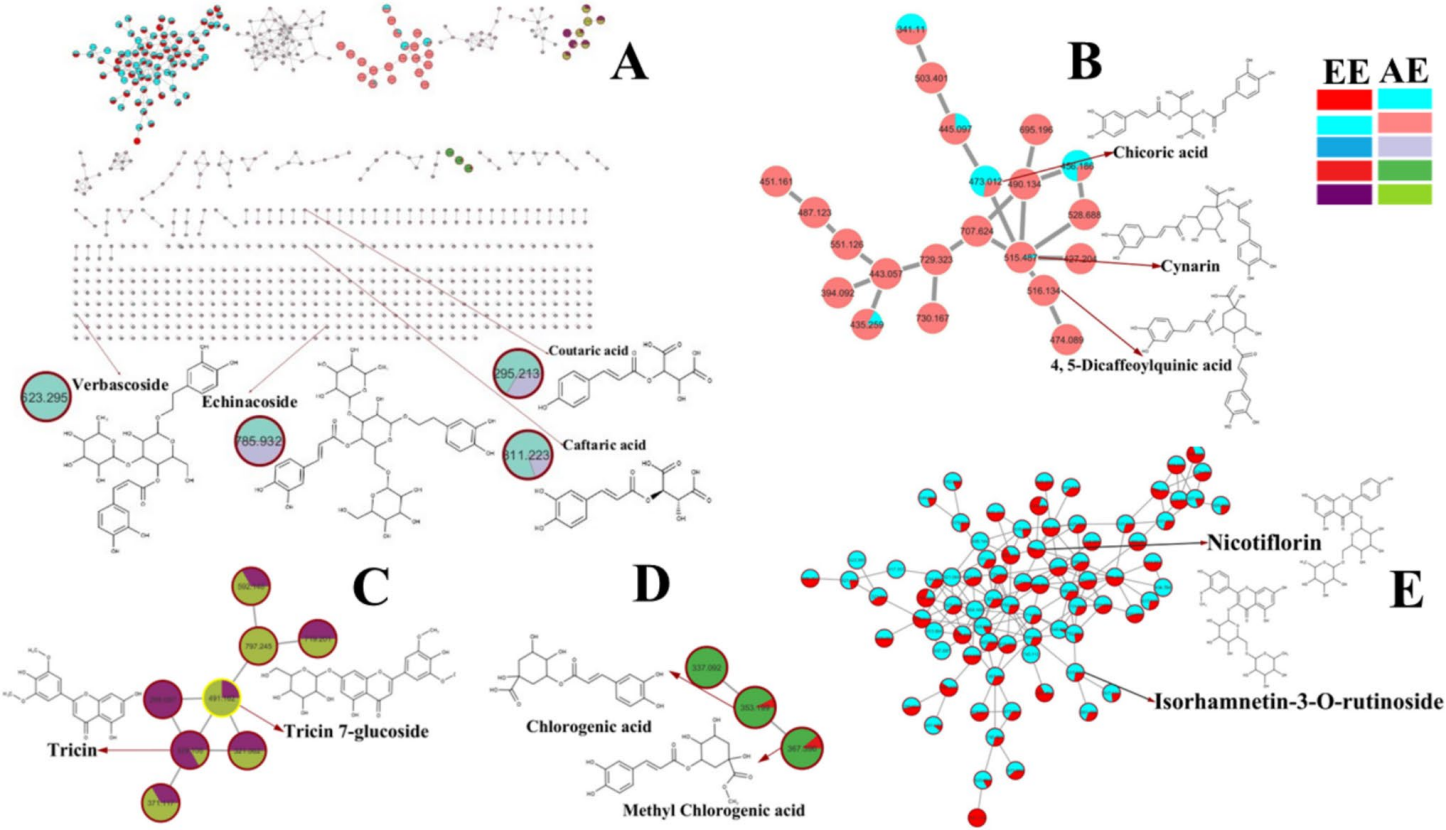
Molecular networking and identification of secondary metabolites from ethanol and aqueous extracts of *E. purpurea* flowers. (A) Molecular networking and chart of categorized compounds in this study. (B) and (D) Zoomed-in molecular networking of phenolic acids. (C) and (E) Zoomed-in molecular networking of flavonoids and flavonoid-*O*-glycosides

The molecular network (negative MS mode) has 698 nodes forming 65 clusters (minimum 2 linked nodes) and 344 self-looped nodes. Each metabolite was represented as a node labeled with its *m*/*z* value. The clusters were generated by connecting groups of metabolites based on the similarity of their fragmentation patterns. (Wang et al. 2016). From molecular networking (Fig. 2), the major clusters included phenolic acids (Fig. 2 B and D), flavonoid derivatives (Fig. 2 C and E), and the self-looped nodes contain phenylethanoid glycosides and other identified metabolites. All nodes were presented as a pie chart to indicate the semi-relative abundance of the detected molecular ions in the tested extracts.

### Evaluation of antioxidant activity

#### DPPH radical scavenging method

The IC_50_ values of EE and AE are 96.05 ± 7.8 and 224.4 ± 17 μg/ml, respectively. The lower value of IC_50_ is considered to represent greater antioxidant activity. The antioxidant potential of the EE was higher than that of AE.

### Ferric reducing antioxidant power assay (FRAP)

The extracts displayed a FRAP 489.57 ± 8.70 for EE and 30.37 ± 2.73 µM Trolox equivalent/mg extract for AE. The FRAP value of EE is higher than that of AE, indicating that EE has a greater capacity to donate electrons than AE.

### Evaluation of *in-vivo* activity of *E. purpurea* extracts

#### E. purpurea extract accelerated wound healing

**Fig. 3:**
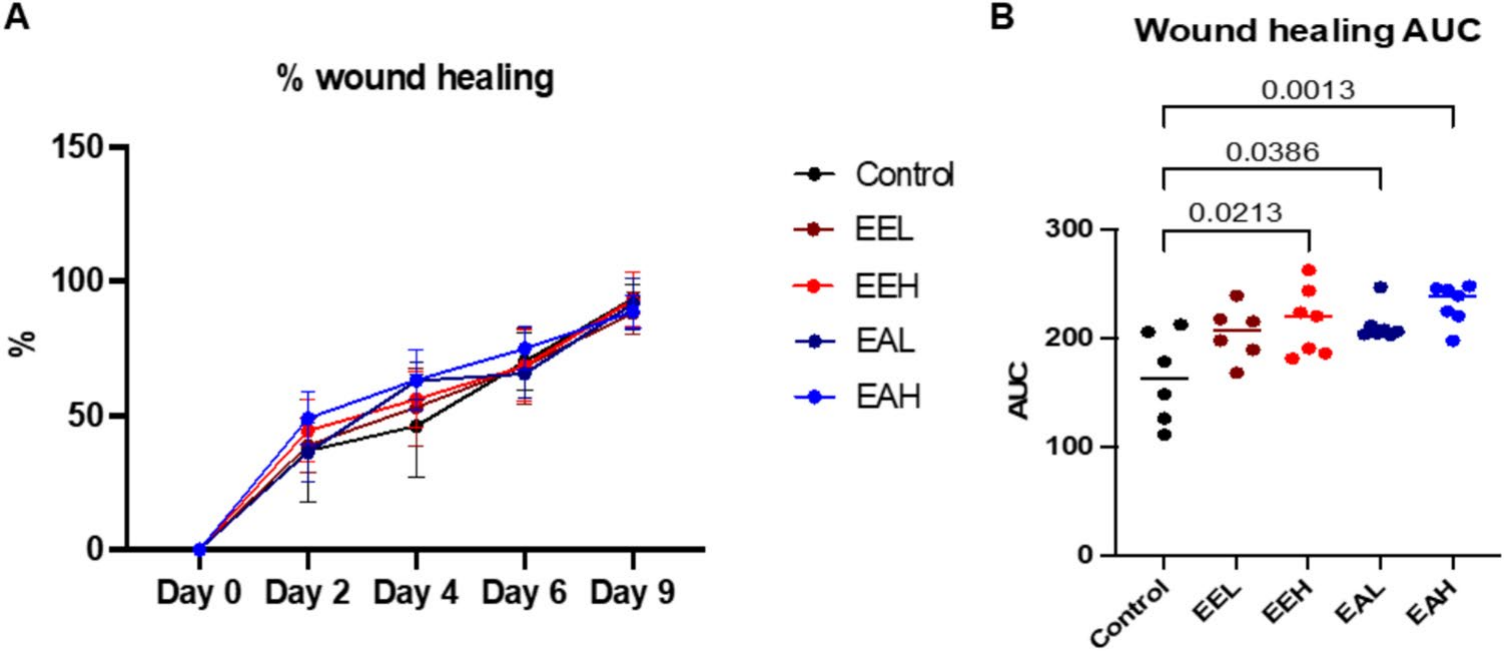
(A) Quantitative analysis of wound size over the experiment time from day-0 (induction day) until day-9. (B) comparison of wound healing rate using area under the curve (AUC) from graph A between control, EEL, EEH, EAL and EAH groups. Data are presented as mean ± SD. Significant difference is considered at P < 0.05

The wounds treated with *E. purpurea* extracts showed a faster rate of healing compared to the control non-treated wounds as shown in Fig. 3A. Moreover, the calculated area under the curve for EEH, EAL and EAH was statistically significantly higher when compared to the control group (P = 0.02, 0.03 and 0.001 respectively, Fig. 3B).

### Histopathology, wound healing criteria and collagen content in wound area

Control group exhibited signs of delayed wound healing represented by incomplete re-epithelization with presence of necrotic debris covering the skin surface, the wound gap was filled with collagen-deficient fibrovascular tissue with intense inflammatory cells infiltration and frequent minute areas of hemorrhage. Generally, *E. purpurea* treated groups with both extract types at different doses showed improvement. Ethanol extract (**EE**) in both doses resulted in enhanced wound healing. Examination of the wound of the ethanol extract treated groups showed complete re-epithelization, reduced inflammation, and wound contraction by formation of collagen rich tissue filling the wound area. Concerning aqueous extract (**EA**) treated groups, the wound healing was also improved in comparison to the control group especially in the terms of good quality organized tissue formation and angiogenesis.

**Fig. 4:**
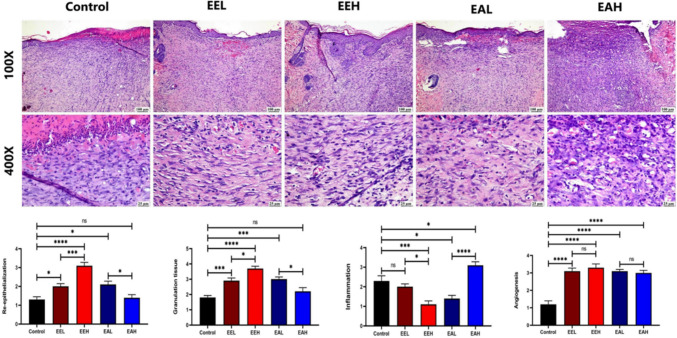
Photomicrographs of skin (H&E) showing wound area in different experimental groups. Charts represent the wound healing scores. Data are presented as mean ± SE. Significant difference is considered at P < 0.05

Regarding the histologic scores of wound healing, generally, the best scores were recorded in **EEH** group followed by the **EEL** group. Both **EA** treated groups showed either significant or numerical improvement when compared to the control group (Fig. 4).

**Fig. 5:**
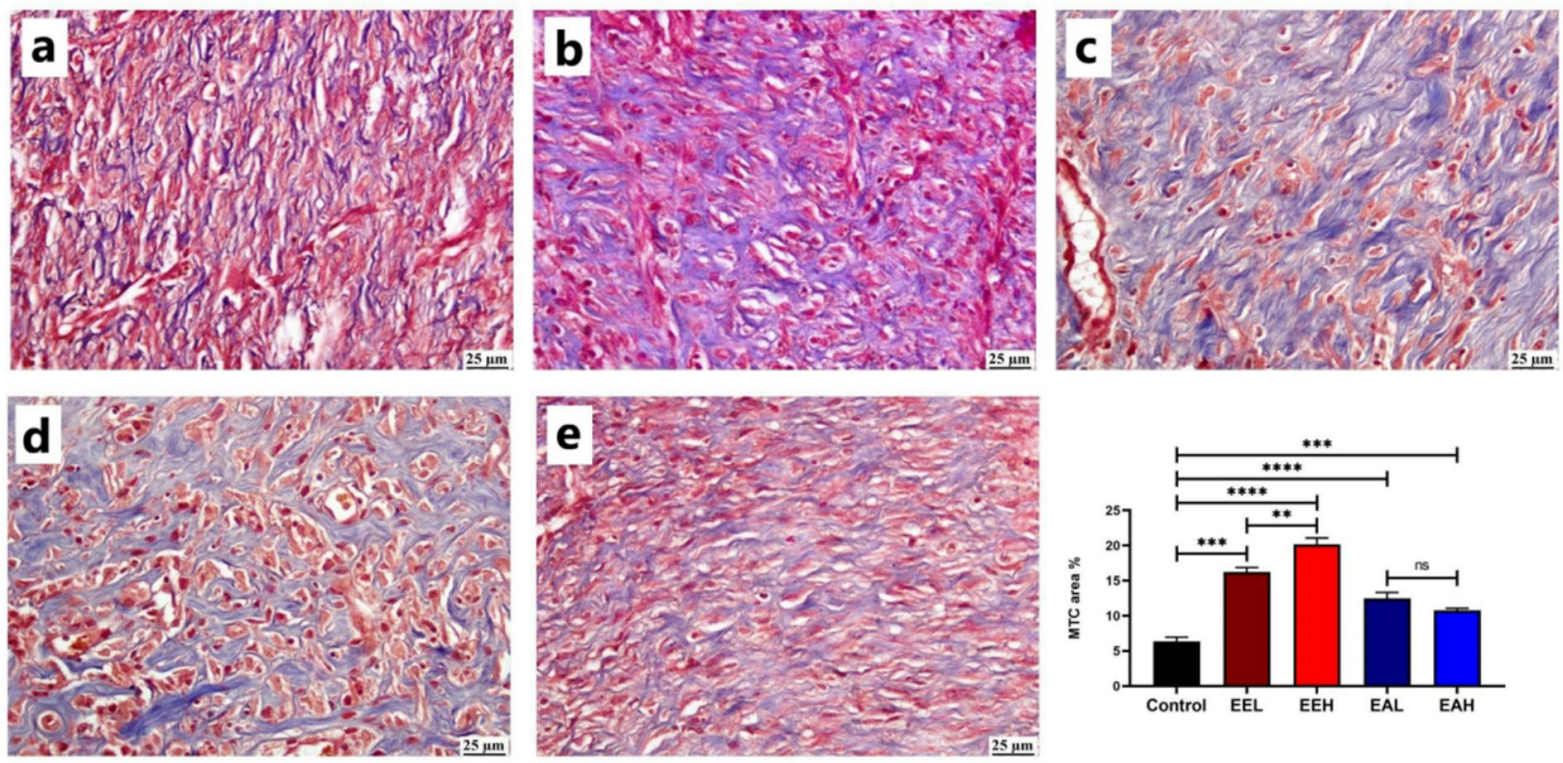
Photomicrographs of skin (MTC) showing collagen content (blue stained) of the wound area in different experimental groups (a) Control, (b) EEL, (c) EEH, (d) EAL, (e) EAH group. Chart represents quantification of blue stained collagen as area percentage. Data are presented as mean ± SE. Significant difference is considered at *P* < 0.05

As illustrated in Fig. 5, **EE** treated groups exhibited highest significant elevation in collagen content of the wound area in a dose dependent manner. **EA** treated groups also showed significant increase in collagen fibers formation with an absence of significance between the two used doses.

### Inflammation and angiogenesis in wound healing

**Fig. 6:**
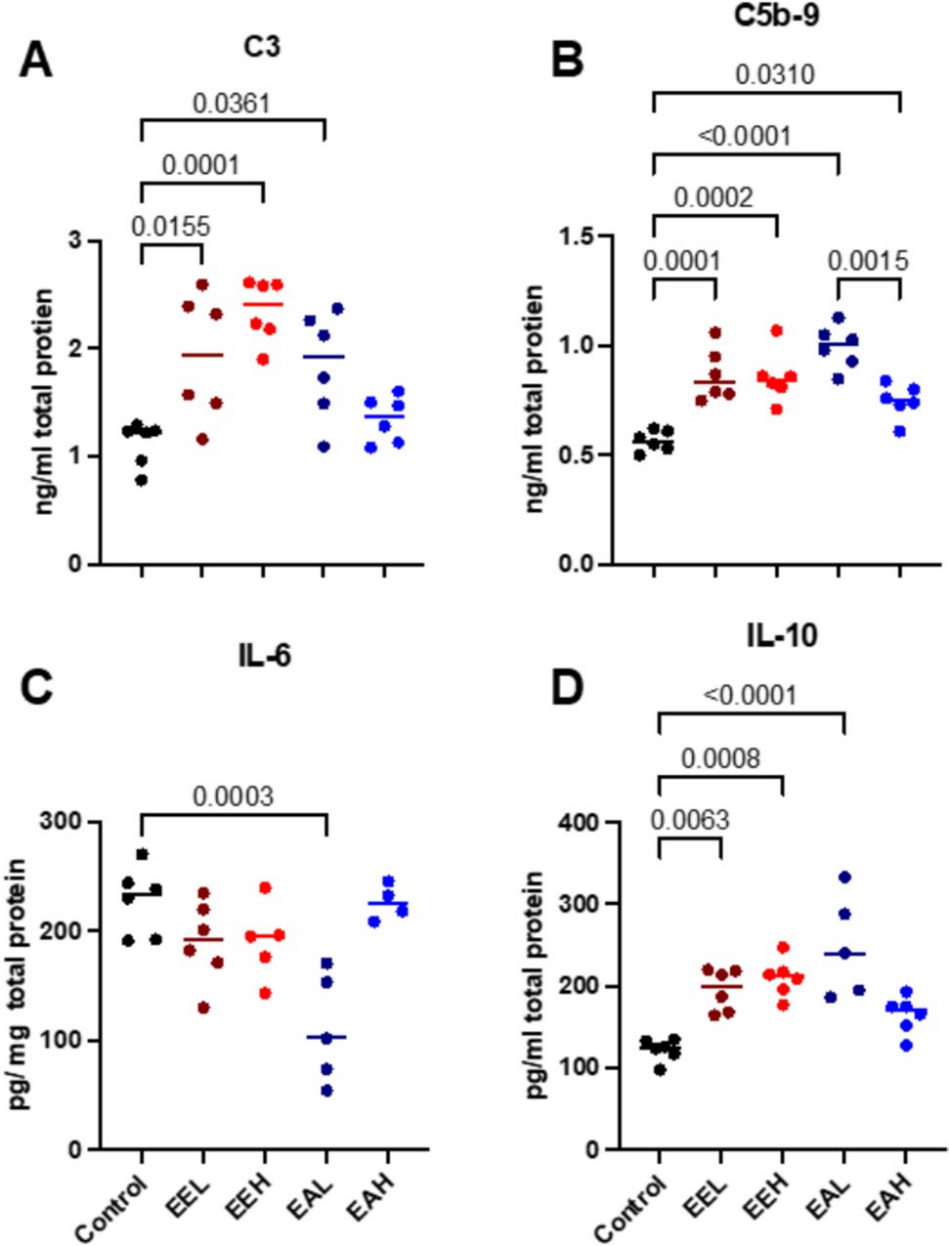
Quantitative analysis of (A) C3, (B) C5b-9, (C) IL-6 and (D) IL-10 in the healed wounded tissue at day 9. Data are represented as scattered plot as mean ± SE. Significant difference is considered at P < 0.05

The immunomodulation effect of the *E. purpurea* on the innate immunity of the healed tissue has been occurred in the statistically significant increase of C3 in the **EEH**, **EEL** and **EAL** compared to the control group (*p* = 0.0001, 0.015 and 0.036 respectively) while there was no difference between **EAH** and control. While C5b-9 proteins expressed of the healed tissue showed the highest difference with **EAL** when compared to control (*p* < 0.0001) and a statistical increase with the other treated groups when compared to the control as shown in Fig. 6A and B, respectively.

The measurement of the IL-6 in the healed tissue was significantly lower in the EAH group when compared to control (p = 0.0003, Fig. 6C) with lower but not significant difference between the control and the other treated groups. While the anti-inflammatory IL-10 showed a statistical higher expression in **EEL**, **EEH,** and **EAL** groups (*p* = 0.0063, 0.0008 and < 0.0001, respectively) compared to the control group with no difference to **EAH** as shown in Fig. 6D.

**Fig. 7:**
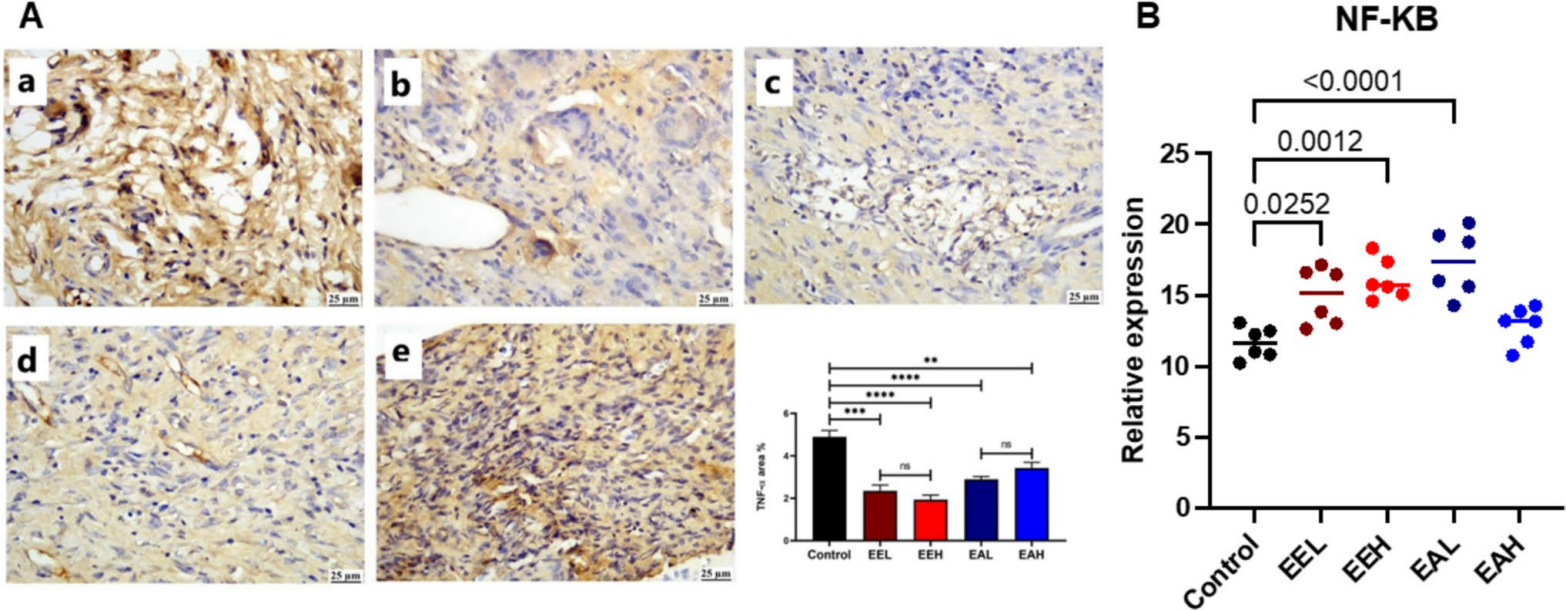
**(A)** Photomicrographs of skin (immunostaining) showing TNF-α in different groups (a) Control group, (b) EEL, (c) EEH, (d) EAL, (e) EAH group. The chart represents TNF-α quantification (as area percentage). (B) Quantitative analysis of NF-kB in the healed wounded tissue at day 9. Data are presented as mean ± SE. Significant difference is considered at P < 0.05

The *E. purpurea* extracts succeeded in downregulating the level of TNF-α release at the healed wound area. **EEH** treated groups showed the least levels of TNF-α followed by those detected in **EA** treated groups (Fig. 7A). The NF-κB was detected in the healed tissue. All the treated groups except **EAH** showed a statistically significant increase compared to the control group as shown in Fig. 7B.

**Fig. 8:**
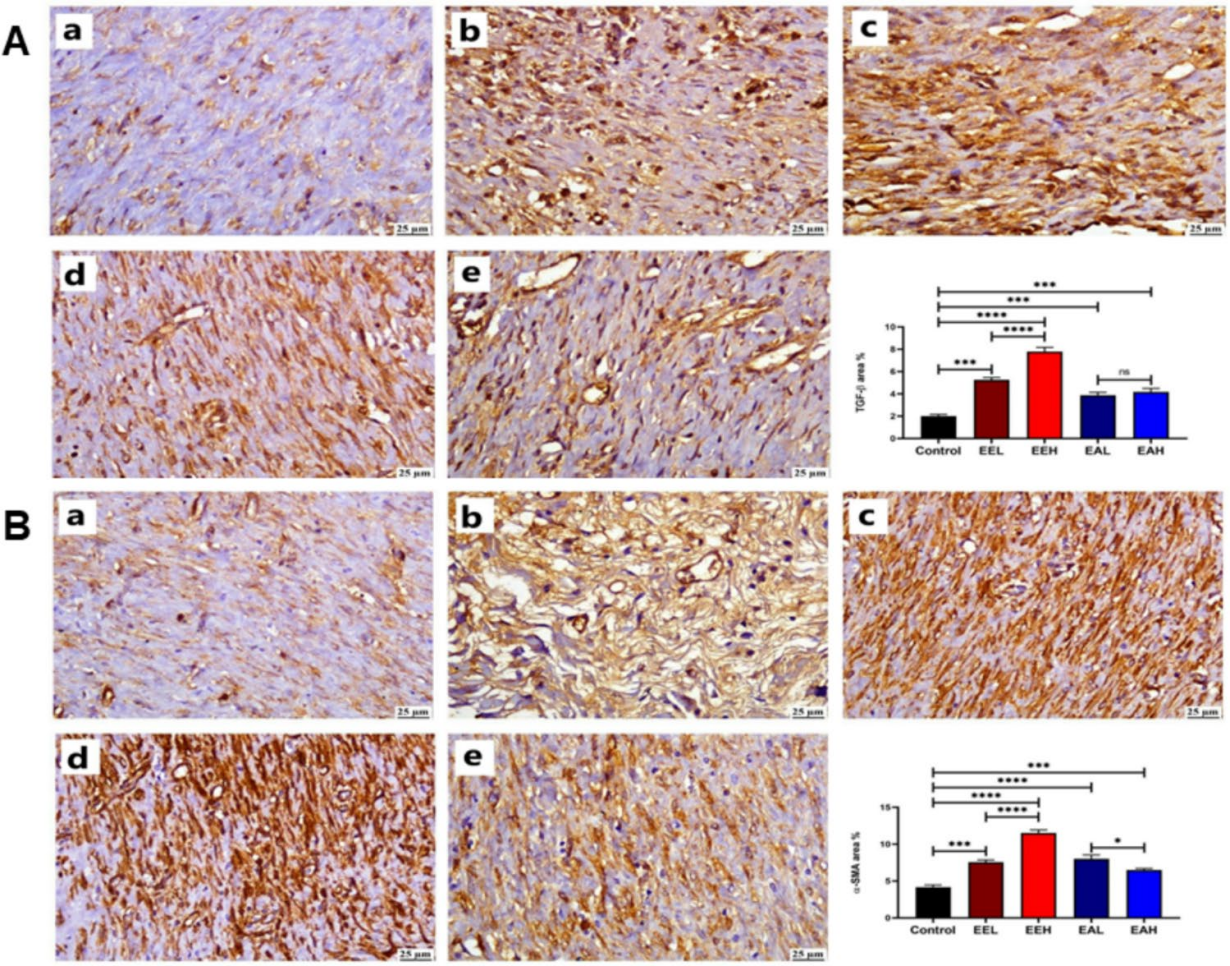
Photomicrographs of skin (immunostaining) showing (A) TGF-β and (B) α-SMA in different groups (a) Control group, (b) EEL, (c) EEH, (d) EAL, (e) EAH group. The chart represents TGF-β and α-SMA quantification (as area percentage). Data are presented as mean ± SE. Significant difference is considered at P < 0.05

Positive expression of TGF-β was markedly increased in the healed wound area of *E. purpurea* treated groups. **EE** extract treated groups showed highest levels of TGF-β in a dose dependent manner followed by that detected in **EA** treated groups. The control group exhibited the lower value of TGF-β as shown in Fig. 8A. Significantly increased α-SMA expression was detected in all treated groups in comparison to the control group. **EA** treated groups exhibited the greatest significant elevation in α-SMA positive staining at the wound area in dose dependent manner (Fig. 8B).

**Fig. 9:**
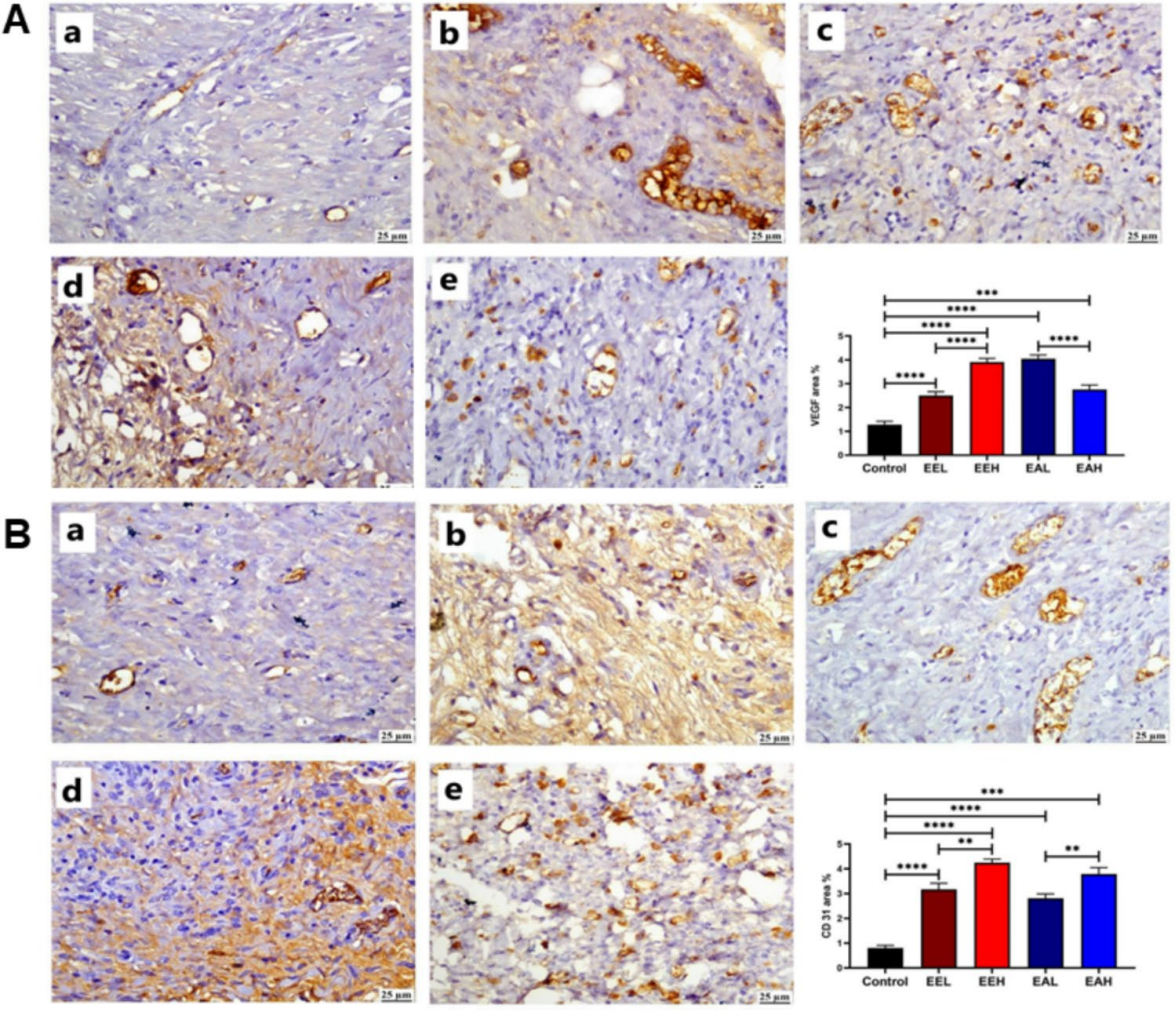
Photomicrographs of skin (immunostaining) showing (A) VEGF and (B) CD-31 in different groups (a) Control group, (b) EEL, (c) EEH, (d) EAL, (e) EAH group. The chart represents VEGF and CD-31 quantification (as area percentage). Data are presented as mean ± SE. Significant difference is considered at P < 0.05

The estimated levels of VEGF were generally enhanced in *E. purpurea* treated groups when compared to the control untreated group. **EE** treatment resulted in highest levels of VEGF in dose dependent manner (Fig. 9A). As illustrated in Fig. 9B, in comparison to the control group, *E. purpurea* treated wound tissue showed significant elevation in CD31 expression in both tested extracts.

## Discussion

The current study focused on the role of ethanol and aqueous extracts of *E. purpurea* on the wound healing model. The UHPLC-MS analysis of EP extracts revealed that polyphenolic compounds were the major identified compounds. Among the identified phenolic compounds, flavonoids and hydroxycinnamic acids derivatives were the most abundant. Chicoric acid, chlorogenic acid, caftaric acid, cynarin and echinacoside were identified in both extracts. Quercetin, kaempferol, isorhamnetin, and their derivatives were among the identified flavonoids. Several studies have proven the wound-healing properties of flavonoids because of their well-known antioxidant, angiogenesis, re-epithelialization, and anti-inflammatory actions (Zulkefli et al. 2023). Echinacoside promotes keratinocyte migration and proliferation, promotes angiogenesis, and activates neutrophils and macrophages, all of which have potential benefits for wound healing (Huang et al. 2022). Phenolic acids, phenylethanoid glycosides, and flavonoids are reported to hasten the process of wound healing which is mainly attributed to their antioxidant, and anti-inflammatory properties (Carvalho et al. 2021; Guimarães et al. 1230).

Antioxidant activity tests showed that EE had significantly more antioxidant activity than AE. This result can be explained by EE’s richness in polyphenolic compounds with high antioxidant activity.

Ethanol is less polar than water, so it can effectively extract a wider range of polar and nonpolar antioxidant compounds from plant materials (Hamdi et al. 2018). This might include a broader spectrum of phenolic compounds, flavonoids, and other antioxidants. Furthermore, ethanol stops the oxidative degradation of chicoric acid, echinacoside, and cynarin by the polyphenol oxidase enzyme, as described by Bergeron et al. 2002; Nüsslein et al. 2000; Wölkart et al. 2004; Skopińska-Różewska et al. 2008).

The use of EE and EA extract at 5% and 10% w/w succeeded in increasing the rate of wound-healing over 9 days of the treatment when applied three times on the wound area. Wounds remain a universal burden on health care and the establishment of an available and affordable treatment in the market can reduce that burden as reviewed before (Freedman et al. 2023).

The innate immunity is directly activated after injury as the first line defense mechanism to prevent wound contamination and infections which delays the healing process (Sinno and Prakash SJPsi. 2013). The use of the Complement C3 and C5 combination in the dressing of the rat wound healing model improves the strength and the rate of healing (Sinno et al. 2013). Interestingly, the current study found that the main complement component C3 in the healed tissue was higher in all the EP treated group with the exception of EAH. Also, the down-stream membrane attack complex was elevated in all the EP-treated groups when compared to the control group. Controversially, Stavros Rafail et al., 2015, reported that complement activation delayed wound healing in a mouse model of wound healing. Where C3 and C5R knockout mice showed an improvement of the rate of wound healing in comparison to their wild type control (Rafail et al. 2015). Recently, Denzinger M et al., 2020 demonstrated in an in-vitro model of wound healing that the increase of sC5b-9 does not reduce re-epithelization and keratinocyte migration/proliferation (Denzinger et al. 2020). Similarly, in the current study, the re-epithelization and granulation were higher in the healed tissue of the EP treated groups except in the EAH group. The EE groups showed improvement in the histopathological examination in a dose-dependent manner while increasing EA concentration showed a reduced healing ability in comparison to other treated groups. The activated complement system induces the early phase of inflammation in order to start wound repair sequences as described by Boniakowski AE et al., 2017 (Boniakowski et al. 2017) and has to subside quickly to allow proliferation, angiogenesis and prevent delayed healing or conversion into a chronic wound as described in a porcine model of wound healing (Holzer-Geissler et al. 2022). In agreement, the histopathological examination of the current study showed reduced inflammation in the healed tissue treated with EE in a dose dependent manner as well as the EAL. Comparably, the pro-inflammatory cytokines IL-6 and TNF-α release were reduced in the healed tissue with an increase of the anti-inflammatory cytokine IL-10. The previous results are in compatible with the Pereira Beserra, F.et al., 2020 where the use of luopeal topical application in a rat model of wound healing reduced the pro-inflammatory cytokine, increased IL-10 and facilitated the rate of wound healing (Pereira Beserra et al. 2020).

The early inflammatory phase is important to induce the NF-κB which an important fine tuning regulator for the balance between fast and delayed repair via its role in modulating immune and inflammatory responses during healing (Ambrozova et al. 2017). In the current study, DNA expression of NF-κB was increased in all the EP treated groups except the EAH group resulting in enhanced the expression of TGF-β in the EE treated groups in a dose dependent manner but not EA treated groups. Furthermore, a similar pattern was seen in the EA treated groups regarding VEGF which is promoted by TGF-β. This agrees with the rat wound healing model treated with phenytoin reported by Savari R et al., 2021 where the gene expression of TGF-β and VEGF increased along with the increase of healing rate (Savari et al. 2019). The clinical manifestation of VEGF is demonstrated as increased angiogenesis in the histopathological examination of all the EP treated groups as well as higher CD31 expression in the newly formed blood vessels in the healed tissue when treated with EE or EA. TGF-β is not only important to start angiogenesis but also α-SMA. TGF-β is important to promote the switch of fibroblasts into myofibroblast and increase α-SMA expression at the wound site as illustrated in the rat wound healing model treated with povidone–iodine (Wang et al. 2017) or mesenchymal stem cells (Putra et al. 2020). This is in accordance with the current study, where different EP extract increased α-SMA expression in the healed tissue. α-SMA is important for healthy healing by enabling extracellular matrix remodeling to start the longer phase of the healing process and stimulate collagen deposition (Urabe et al. 2024). The pattern of collagen deposition of the EP treated healed tissue was explained by the α-SMA expression.

## Conclusion

In conclusion, the ethanol extract obtained from the flower of *E. purpurea* is a good source of many bioactive metabolites, including flavonoids, phenolic acids, and phenylethanoid glycosides, and showed a beneficial effect on the wound healing process and in a dose-dependent manner whereas the high dose of aqueous extract was far less effective. The findings strongly suggest that the ethanol extract may be worth further investigation for potential applications in topical treatment related to wound healing.

## Declarations

### Data availability

The authors declare that the data supporting the findings of this study are available within the paper. Should any raw data files be needed in another format they are available from the corresponding author upon reasonable request.
